# **α**-Mangostin from *Cratoxylum arborescens* (Vahl) Blume Demonstrates Anti-Ulcerogenic Property: A Mechanistic Study

**DOI:** 10.1155/2013/450840

**Published:** 2013-03-24

**Authors:** Heyam M. A. Sidahmed, Siddig Ibrahim Abdelwahab, Syam Mohan, Mahmood Ameen Abdulla, Manal Mohamed Elhassan Taha, Najihah Mohd Hashim, A. Hamid A. Hadi, Jamunarani Vadivelu, Mun Loke Fai, Mawardi Rahmani, Maizatulakmal Yahayu

**Affiliations:** ^1^Department of Pharmacy, Faculty of Medicine, University of Malaya, 50603 Kuala Lumpur, Malaysia; ^2^Medical Research Centre, Jazan University, P.O. Box 114, Jazan, Saudi Arabia; ^3^Department of Molecular Medicine, Faculty of Medicine, University of Malaya, 50603 Kuala Lumpur, Malaysia; ^4^Department of Chemistry, Faculty of Science, University of Malaya, 50603 Kuala Lumpur, Malaysia; ^5^Department of Medical Microbiology, Faculty of Medicine, University of Malaya, 50603 Kuala Lumpur, Malaysia; ^6^Department of Chemistry, Faculty of Science, Universiti Putra Malaya, 43400 Serdang, Malaysia

## Abstract

*Cratoxylum arborescens* (Vahl) Blume is an Asian herbal medicine with versatile ethnobiological properties including treatment of gastric ulcer. This study evaluated the antiulcerogenic mechanism(s) of **α**-mangostin (AM) in a rat model of ulcer. AM is a prenylated xanthone derived through biologically guided fractionation of *C. arborescens*. Rats were orally pretreated with AM and subsequently exposed to acute gastric lesions induced by ethanol. Following treatment, ulcer index, gastric juice acidity, mucus content, histological and immunohistochemical analyses, glutathione (GSH), malondialdehyde (MDA), nitric oxide (NO), and nonprotein sulfhydryl groups (NP-SH) were evaluated. The anti-*Helicobacter pylori*, cyclooxygenase-2 (COX-2) inhibitory effect, and antioxidant activity of AM were also investigated *in vitro*. AM (10 and 30 mg/kg) inhibited significantly (*P* < 0.05) ethanol-induced gastric lesions by 66.04% and 74.39 %, respectively. The compound induces the expression of Hsp70, restores GSH levels, decreases lipid peroxidation, and inhibits COX-2 activity. The minimum inhibitory concentration (MIC) of AM showed an effective *in vitro* anti-*H. pylori* activity. The efficacy of the AM was accomplished safely without presenting any toxicological parameters. The results of the present study indicate that the antioxidant properties and the potent anti-*H. pylori*, in addition to activation of Hsp70 protein, may contribute to the gastroprotective activity of **α**-mangostin.

## 1. Introduction

Peptic ulcer disease (PUD) is the most common gastrointestinal tract aliment of multiple causes which hinders a lot of people worldwide. PUD occurs when the offensive factors overcome the defensive ones [1, 2]. There are many noxious agents that attack the stomach resulting in mucosal ulceration such as* Helicobacter pylori *infection, excessive ingestion of nonsteroidal anti-inflammatory drugs (NSAIDs), or alcohol as well as psychological stress and cigarette smoking. On the other hand, the stomach protects itself by many defensive mechanisms, mainly the entire mucosal layer which acts as a barrier against inflammatory and cytotoxic agents [3]. It is reported that accumulation of reactive oxygen species is in charge of peptic ulcer aetiology in various gastric ulcer models suggesting the participation of antioxidant enzymes, and therefore there is a trend to explore antioxidant drugs from natural resources [2]. The reciprocal antiulcer medication to treat the peptic ulcer is either by inhibiting gastric acid secretion or by enhancing mucus layer protection. However, majority of them possess adverse effects that may limit their usage [4]. For that there is need to discover new antiulcer drugs. Recent studies have proven a variety of antiulcer compounds of botanical origin which mainly are alkaloids, saponins, xanthones, triterpenes, and tannins compounds [1].

The chemical constituents of Cratoxylum species is not well studied so far regardless of their popular traditional uses. However, recent studies on this genus have led to the isolation and identification of oxygenated and prenylated xanthones and some anthraquinones. *Cratoxylum arborescens* is a well-known tropical tree. The bark, roots, and leaves of this plant have been used in folk medicine to treat fever, coughs, diarrhea, itches, ulcers, and abdominal complaints. The plant was reported to possess cytotoxic effect against human lung cancer cell line (NCI-H187) with IC_50_ values ranging from 0.65 to 5.2 *μ*g/mL [5, 6]. Upon these potential activities, *C. arborescens* was subjected to several experimental tests to evaluate its antiulcerogenic property and to identify possible gastroprotective mechanism(s) of its major bioactive compound against ethanol ulcer models in experimental animals.

## 2. Material and Methods

### 2.1. Extraction and Isolation of *α*-Mangostin (AM) from *C. arborescens*

**Figure 1:**
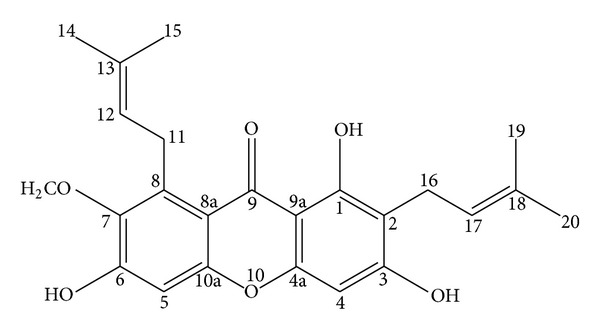
Chemical structure of *α*-mangostin from *Cratoxylum arborescens* (Vahl) Blume.

The stem bark of *C. arborescens* was collected from wild trees growing in Sarawak, Malaysia, in 2009. A voucher specimen has been deposited at the Herbarium, Department of Biology, Faculty of Medicine, Universiti Putra Malaysia. The finely ground air-dried stem bark of *C. arborescens* (1.0 kg) was extracted consecutively with hexane, chloroform, and methanol to give 6.12, 28.18, and 40.27 g, respectively, of dark viscous semisolid material on solvent removal. The hexane extract was chromatographed over vacuum column and eluted with solvent of gradually increasing polarity to give 26 fractions of 200 mL each. After extensive fractionation and purification of fractions 14–20 yielded *α*–mangostin (AM, Figure 1). 

### 2.2. Identification of *α*-Mangostin

Melting point of AM was 178–180°C ([7], m.p 181-182°C). UV MeOH *λ*_max⁡_ nm (log *ε*): 390 (2.41), 358 (3.99), 316 (3.99), and 238 (2.65). IR *ν*_max⁡_ cm^−1^ (KBr): 3369 (OH), 2934 (CH), 1608 (C=C), 1462, and 1286. EIMS *m/z* (%intensity): 410 (43.06), 395 (6.14), 379 (1.61), 354 (25.77), 339 (100.00), 311 (32.57), 296 (12.89), 285 (18.90), 257 (6.46), and 162 (14.16). ^1^H-NMR (500 MHz, acetone-*d*_6_): *δ* 13.79 (OH-1), 9.62 (OH-6), 9.52 (OH-3), 6.81 (*s*, 1H, H-5), 6.38 (*s*, 1H, H-4), 5.26 (*t*, *J* = 6.85 Hz, 2H, H-12, and H-17), 4.12 (*d*, *J *= 6.85 Hz, 2H, H-11), 3.78 (OMe-7), 3.35 (*d*, *J* = 8.00 Hz, 2H, H-16), 1.82 (*s*, 3H, Me-14), 1.71 (*s*, 3H, Me-19), and 1.64 (*s*, 6H, Me-15, and Me-20). ^13^C-NMR (125 MHz, acetone-*d*_6_): *δ* 182.0 (C-9), 162.1 (C-4a), 160.9 (C-1), 156.6 (C-10a), 155.4 (C-6), 154.9 (C-3), 143.6 (C-7), 137.3 (C-8), 130.6 (C-18 and C-13), 123.9 (C-12), 122.6 (C-17), 111.2 (C-8a), 110.2 (C-2), 102.8 (C-9a), 101.9 (C-5), 92.3 (C-4), 62.5 (OMe-7), 26.1 (C-11), 25.1 (C-15 and C-20), 21.1 (C-16), 17.5 (C-14), and 17.1 (C-19). Purity of AM was checked and confirmed using HPLC and LC/MS. 

### 2.3. Drugs and Reagents

Griess reagent, omeprazole, DTNB (2,2′-dinitro-5,5′-dithiodibenzoic acid), and TPTZ (2, 4, 6-tri (2-pyridyl)-1,3,5-triazine) were purchased from Sigma-Aldrich (Singapore). All other used chemicals and reagents were of analytical grade.

### 2.4. Acute Toxicity Study

Adult male and female ICR mice (6–8 weeks old: 20–30 g) were obtained from the Experimental Animal House (Ethic No. PM 07/05/2008 MAA (a)(R)), Faculty of Medicine, University of Malaya. Thirty animals were assigned equally into three groups. Animals were fasted (24 h) prior to oral administration of AM at doses of 30 and 300 mg/kg b.w., respectively. Third group of mice were administered with 5% Tween80 and served as a control group. Animals were observed for two weeks for any mortality or changes, particularly changes in skin, eyes, mucus membrane, and also autonomic and central nervous system. Food and water were provided throughout the experiment *ad libitum*. On day 15th animals were sacrificed, the blood collected for biochemical parameters analysis and the organs (heart, lungs, spleen, liver, and the kidney) were excised for organ weight variation and histology study. All animals received human care according to the criteria outlined in the “*Guide for the Care and Use of Laboratory Animals*” prepared by the National Academy of Sciences and published by the National Institute of Health, Malaysia.

### 2.5. Induction of Acute Gastric Lesion

Sprague Dawley male rats (220–250 g) were obtained from the Experimental Animal House (Ethic No. PM 07/05/2008 MAA (a)(R)), Faculty of Medicine, University of Malaya. To avoid coprophagy, rats were kept individually in cages with raised floors of wide mish and divided randomly into four groups (*n* = 6). The animals were fasted for 48 h prior to oral dosing. One h before intragastric administration of ethanol (5 mL/kg) animals were orally pre-treated as follows: group (1) served as normal control without treatment, group (2) treated with vehicle (5% Tween80, v/v, 5 mL/kgb·w.), group (3) omeprazole (20 mg/kg), and group (4 and 5) with AM (10 and 30 mg/kg, resp.). One h after ethanol dosing, all animals were sacrificed under anesthesia (ketamine and xylazine), and their blood was collected [2]. 

#### 2.5.1. Measurement of Gastric Juice Acidity, Mucus Content, and the Biochemical Parameters

Stomach contents of each animal were collected and centrifuged to measure the concentration of the hydrogen ion concentration (pH) from the supernatant using the pH meter expressed as meq/l and the weight of the gastric mucosa from the sedimentation using precise balance. Animal Blood samples were analyzed at University Malaya Medical Centre, Kuala Lumpur, Malaysia, to evaluate changes in serum biomarkers [8]. 

#### 2.5.2. Gastroprotective Assessments

Gastric ulcer appears as elongated bands of hemorrhagic lesions. The length (mm) and the width (mm) of each band were measured using planimeter ((10 × 10 mm 2 = ulcer area) under dissecting microscope (1.8×)). The area of each ulcer lesion was measured by counting the number of small squares (2 mm × 2 mm) covering the length and width of each ulcer band. The sum of the areas of all lesions for each stomach was applied in the calculation of the ulcer area (UA) where in the sum of small squares × 4 × 1.8 = UA mm^2^. The inhibition percentage (I%) was calculated by the following formula described in [9] with slight modifications:

(1) Inhibition percentage(I%) =[(UA control−UA treated)UA control]×100%.

#### 2.5.3. Histopathological Evaluation Using Hematoxylin and Eosin, Periodic Acid-Schiff, and Immunohistochemistry

For histopathological evaluation, a small fragment of each animal gastric ulcer was fixed with 10% buffered formalin solution. Formalin-fixed tissues were dehydrated with alcohol and xylene and finally embedded in paraffin wax. Tissue sections of 5 *μ*m were stained with hematoxylin and eosin (H and E) for light microscopy [10]. Slides were also stained with Periodic Acid-Schiff (PAS) Base [11], for the evaluation of mucus production, following the manufacturer's instructions (Sigma-Aldrich, Singapore). A primary antibody of HSP-70 (dilution 1 : 500) was used to investigate the mechanism of AM as a potential antiulcerogenic agent. Immunohistochemical detection for these antibodies was done using Animal Research Kit (ARK, DakoCytomation). 

#### 2.5.4. Determination of Glutathione (GSH) Levels

Total GSH content was estimated using the following method [12]. In brief, small fragments of each stomach were homogenized in ice-cooled trichloroacetic acid (5%, w/v). The homogenates were centrifuged at 7000 × g for 15 min at 4°C, and the supernatants were used to quantify GSH content through reaction with DTNB. Absorbance was read in 412 nm, and the results were expressed in nmol GSH/g tissue.

#### 2.5.5. Determination of Thiobarbituric Acid Reactive Substance and Nitric Oxide

Thiobarbituric acid reactive substance (TBARS) assay was used to estimate malondialdehyde (MDA) content according to the method of [13]. Briefly 0.5 mL of the 10% stomach homogenate in 0.1 mol/L PBS was added to a solution containing 1.5 mL acetic acid (20%, w/v), 2.0 mL of 20% sodium dodecyl sulphate, and SDS (pH 3.5, adjusted with NaOH). The mixture was incubated in a water bath at 95°C for 1 h. After cooling, 3 mL of the mixture was vortex and centrifuged at 3000 g for 10 min. The absorbance of the supernatant was determined in a spectrophotometer at 532 nm, and the values of MDA in the gastric tissue are expressed in *μ*mol/g tissue malondialdehyde. Nitric oxide (nmol/g tissue) was quantified in gastric homogenates using the Griess assay [14]. 

#### 2.5.6. Estimation of Nonprotein Sulfhydryls

Gastric mucosal nonprotein sulfhydryls (NP-SH) (*μ*mol/g of tissue) were measured [15]. In brief, an aliquot of 5 mL of the stomach homogenate was mixed with a solution containing 4 mL of distilled water and 1 mL of 50% trichloroacetic acid. The mixture was vortex for 15 min and centrifuged at 3000 × g. Two mL of the supernatant were mixed with 4 mL of 0.4 M Tris buffer (pH 8.9). One hundred microliter of DTNB [5,5 dithiobis-(2-nitrobenzoic acid)] was added. The absorbance was recorded within 5 min of addition of DTNB at 412 nm against a reagent blank with no homogenate. 

### 2.6. *In Vitro* Evaluation of Cyclooxygenase-2 Inhibitory Activity

*α*-Mangostin was tested for its COX-2 inhibitory activity using a COX-inhibitor screening kit according to the manufacturer's instructions (Cayman Chemical, USA). COX-2 enzyme catalyzes the biosynthesis of prostaglandin-H-synthase-2 from arachidonic acid. This assay measures the production of PGF2*α* generated by SnCl_2_, using enzyme immunoassay (acetylcholine esterase (ACE) competitive EIA). The stock solution of AM was dissolved in dimethyl sulfoxide (DMSO), and the final concentration was obtained from 0 to 100 *μ*g/mL using the reaction buffer. Inhibition was calculated by the comparison to control incubations. 

### 2.7. *In Vitro* Antioxidant Assays

The Ferric-reducing antioxidant power (FRAP) assay was accomplished following the procedure described earlier [16] with slight modifications. Briefly, the FRAP reagent was prepared freshly from acetate buffer (pH 3.6), 10 mM TPTZ [2,4,6-Tri(2-pyridyl)-s-triazine] solution in 40 mM HCl and 20 mM iron (III) chloride solution in proportions of 10 : 1 : 1 (v/v), respectively. Fifty *μ*L of the compound were added to 1.5 mL of the FRAP reagent in the dark; 4 min later the absorbance was then recorded at 593 nm. The standard curve was constructed linearly (*R* ^2^ = 0.996) using iron (II) sulfate solution (0–1000 *μ*M), and the results were expressed as *μ*M of Fe(II)/g dry weight of the compound. DPPH was conducted according to the method described by Mahmood et al. [23]. 

### 2.8. *In Vitro* Anti-*Helicobacter Pylori* Activity

Two *H. pylori* strains, NCTC 11637 (ATCC43504), and J99 (ATCC700824) were cultured with brain heart infusion broth supplemented with 10% horse serum (Invitrogen, Malaysia) incubated at 37°C in a humidified CO_2_ incubator for 3 days. Minimum inhibitory concentration (MIC) was determined by a modified microtiter broth dilution method on sterile 96-well polypropylene microtitre plates with round-bottom wells (Eppendorf, Germany). Briefly, AM was dissolved and diluted in 5% DMSO to give a 10x working stock solution. *H. pylori* was diluted to a final concentration of 2 × 10^6^ CFU/mL in culture medium. Aliquots of 10 *μ*L of AM were added to 90 *μ*L of *H. pylori* in a well of the microtitre plate. Concentration of AM ranged from 0 to 250 *μ*g/mL. The microtiter plate was incubated for 3 days in a CO_2_ incubator. The plate was examined visually and measured using a microplate reader (Varioskan Flash) at 600 nm to determine the lowest concentration showing complete growth inhibition, which was recorded as the MIC. Minimum bactericidal concentration (MBC) as the lowest concentration without growth on a chocolate agar plate supplemented with 7% lysed horse blood. Wells containing *H. pylori* with 10 *μ*L of 5% DMSO and BHI medium containing 250 *μ*g/mL AM were used as control and blank, respectively. The result was recorded in accordance with the Clinical and Laboratory Standards Institute [17].

### 2.9. Statistical Analysis

All values were reported as mean ± S.E.M. Statistical significant differences between groups were assessed using one-way ANOVA followed by Tukey's *post hoc *multiple comparison test. A value of *P* < 0.05 or lower was considered as a significant difference. 

## 3. Results

### 3.1. Toxicity Study

There were no abnormal physiological, behavioural changes or body weight alterations at any time point of observation at the doses used during the two weeks. Histological examination to the liver and kidney and serum biochemical analysis did not show any difference in comparison to the control group (data not shown but available upon request).

### 3.2. Antiulcer Study

#### 3.2.1. Gross and Macromorphometric Evaluation

**Figure 2:**
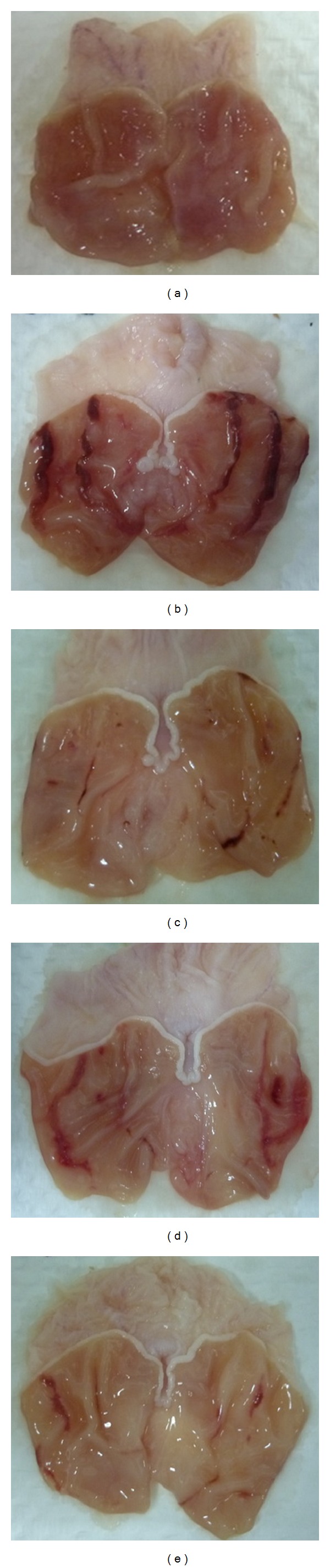
Gross evaluation of stomachs from various animal groups. Results showed that rats pretreated with *α*-mangostin (AM) at doses of 10 and 30 mg/kg ((d) and (e), resp.) and omeprazole ((c); 20 mg/kg) had considerably reduced areas of gastric ulcer formation compared to rats pretreated with only Tween80 (ulcer control group, (a)) (magnification: 1.8x).

Results showed that animals pretreated with AM and omeprazole considerably reduced ulcer area formation compared to animal group pretreated with only 5% Tween80 (Table 1). *α*-Mangostin (AM) at doses of 10 and 30 mg/kg b.w. significantly (*P* < 0.05) inhibited ulcer formation by 66.04% and 74.39%, respectively, as shown in Table 1 and Figure 2.

#### 3.2.2. Gastric Mucus Content, Acidity and Biochemical Analysis

As shown in Table 1, ulcer control group produced the lowest mucus content of gastric mucosa, while 10 and 30 mg/kg of AM increased significantly (*P* < 0.05) the mucus production. On the other hand, animal groups pretreated with AM showed increase in the pH of the gastric contents. Serum analysis showed that rats induced with ethanol ulceration had increased levels of liver enzymes (AST and ALT) compared to normal and AM-treated groups as shown in Table 2.

#### 3.2.3. Morphological Findings

**Figure 3:**
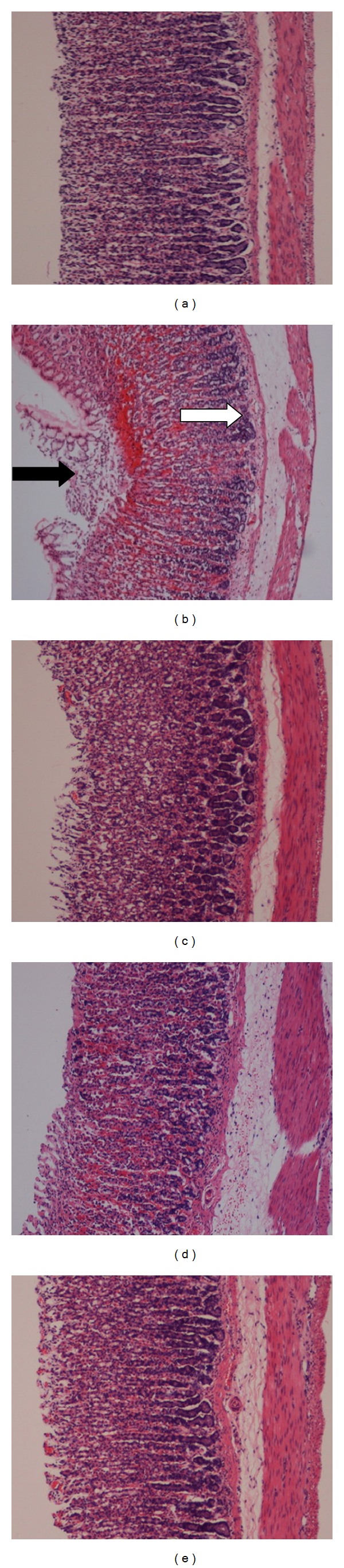
Histopathological evaluation. Results showed that *α*-mangostin (AM) at doses of 10 and 30 mg/kg ((d) and (e), resp.) and omeprazole ((c); 20 mg/kg) improved the histopathology of rats' stomach compared to those pretreated with only Tween80 (ulcer control group, (b)). Black arrow indicates severe disruption to the epithelium surface and deep mucosa, while white arrow indicates leukocyte infiltration and edema in the submucosa layer (H and E stain; magnification: 10x).

**Figure 4:**
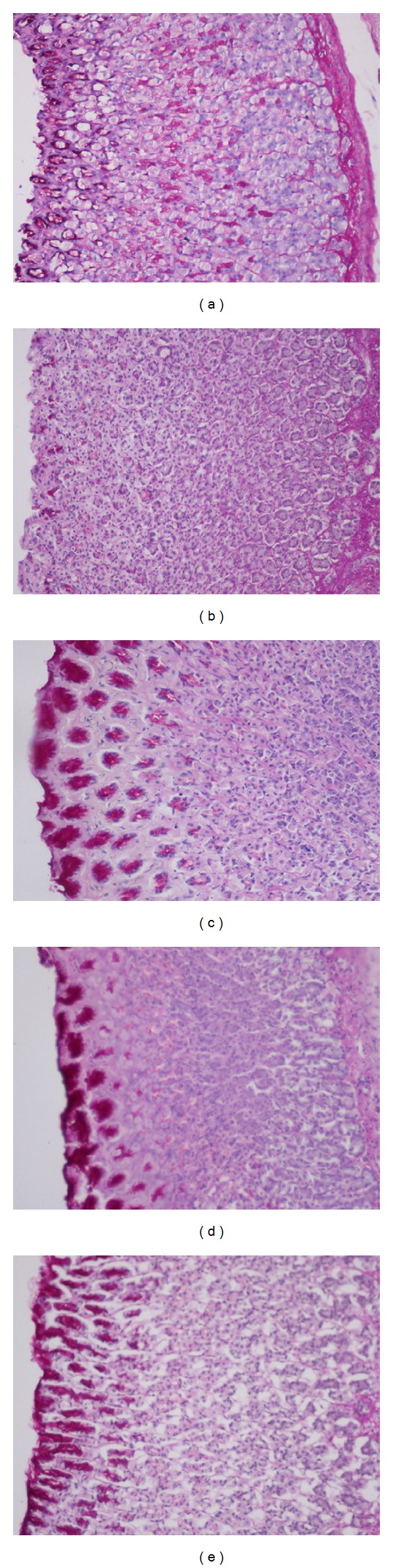
PAS staining for the evaluation of mucus production. Results showed that rats pretreated with *α*-mangostin (AM) at doses of 10 and 30 mg/kg ((c) and (d), resp.) and omeprazole ((e); 20 mg/kg) showed more PAS-positive mucus as compared to those pretreated with only Tween80 (ulcer control group, (b)) (Magnification: 10x).

Histological observation of the ulcer control group pretreated only with 5% Tween80 showed highly extensive gastric lesion, submucosal edema, and leucocytes infiltration. Pretreatment with AM (10, 30 mg) and omeprazole has relatively better protection as seen by decreasing ulcer area, reduction or complete absence of edema, and leucocytes infiltration as shown in Figure 3. Treatment of animals with AM resulted in the expansion of a substantial continuous PAS-positive mucous layer that lining the entire gastric mucosal surface noted as a bright-purple-stained area lining the mucosa as shown in Figure 4.

#### 3.2.4. Protein Expression (HSP-70)

**Figure 5:**
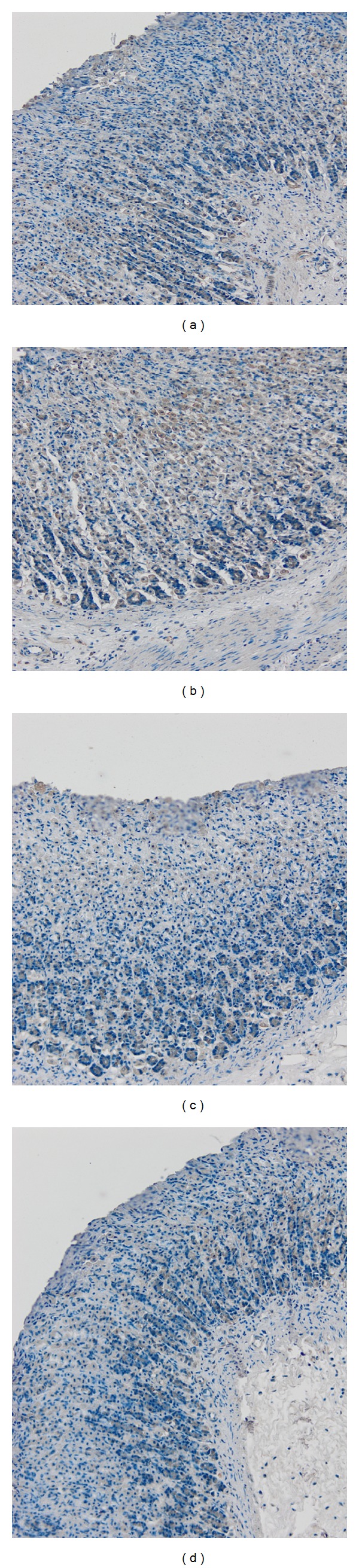
Immunohistochemical staining of rat gastric tissues with Hsp70 primary antibodies. (a) Gastric tissues from AM (10 mg/kg) pretreated animals; (b) gastric tissues from AM (30 mg/kg) pretreated animals; (c) gastric tissues from ethanol-induced animals; (d) gastric tissues from omeprazole (20 mg/kg) pretreated animals (magnification: 10x).

To understand the mechanism of AM as an antiulcerogenic agent, gastric tissues from various experimental groups were stained with immunohistochemistry as shown in Figure 5. The gastric tissues were examined for immunohistochemical localization of Hsp70 protein. Microscopic examination reveals high numbers of proliferated cell as well as modulation of Hsp70. These results indicate an effective role of this antioxidant protein in the gastroprotection mechanism of AM.

#### 3.2.5. Involvement of Oxidative Stress Markers and COX-2

Glutathione levels were significantly (*P* < 0.01) lower in the ulcer control group than the normal group. The treatment of animals with the AM restored significantly (*P* < 0.01) the GSH levels depletion after ethanol administration. The ulcer control group showed the highest level of malondialdehyde (MDA), an indicator of lipid peroxidation, than the other groups. Gastric MDA level significantly (*P* < 0.05) dropped after AM administration. Results for MDA, NO, and GSH are shown in Table 2.

To investigate the gastroprotective mechanism, NP-SH levels were estimated. The compound alleviated NP-SH level which was dropped due to ethanol administration. Moreover, it was observed that AM in two concentrations 250 ng/mL and 500 ng/mL inhibited COX-2 activity *in vitro *by 27.7% and 58.8% in comparison to the standard COX-2 inhibitor, indomethacin (72.6%). 

### 3.3. Antioxidant Evaluation of *α*-Mangostin

*α*-Mangostin exhibited FRAP value of 112.8 ± 0.23, while the positive controls used in this study exhibited 2140.61 ± 0.061, 770.39 ± 0.003, 2460.67 ± 0.027, and 421.94 ± 0.003 for trolox, quercetin, gallic acid, and ascorbic acid, respectively. This natural compound did not show strong radical scavenging activity with no value against DPPH. 

### 3.4. *In Vitro* Anti-*Helicobacter Pylori* Activity

*α*-Mangostin represents minimum inhibitory concentration (MIC) of 24 *μ*g/mL and minimum bactericidal concentration (MBC) of 246 *μ*g/mL against *H. pylori* (Strain no. NCTC11637) and MIC 19 *μ*g/mL and MBC of 124 *μ*g/mL against *H. pylori *(Strain no. J99).

## 4. Discussion 

*Cratoxylum arborescens* is well-known ethnomedicine used in Asia for the treatment and prevention of gastric ulcer. *α*-Mangostin (AM), as a natural product, is a prenylated xanthone isolated by our group through biologically guided fractionation from *C. arborescens*. Therefore, this study evaluated the antiulcerogenic mechanism(s) of AM in a rat model of ulcer. This natural compound was reported earlier to possess many biological properties, such as anti-inflammatory, antioxidative damage, and antioxidant activities [18]. 

Gastric ulcer is caused by different etiologies [19]. It is generally believed that this disease is the consequence of an imbalance between destructive factors and defensive mechanisms of the mucosal integrity [20]. Ethanol as a potential gastroulcerogenic agent is known to be involved in the depression of these defensive mechanisms [21]. The mechanism of this potential gastrotoxic agent includes microvascular injury, increased vascular permeability, edema formation, and epithelial lifting. Ethanol produces necrotic lesions in the gastric mucosa, increased flow of Na^+^ and K^+^, increased pepsin secretion, and loss of H^+^ ions and histamine into the lumen [22]. Ethanol is commonly used for inducing ulcer in experimental rats and leads to intense gastric mucosal damage through oxidative injury. Ethanol produces a marked contraction of the circular muscles of rat fundic strip [23]. Alterations in the gastric motility and relaxation of circular muscles may protect the gastric mucosa. Such contraction can lead to mucosal compression at the site of the greatest mechanical stress, at the crests of mucosal folds leading to necrosis and ulceration [24]. Flattening of the gastric folds will increase the mucosal area exposed to ulcerogenic factors and reduce the volume of the gastric irritants on rugal crest. In the present study, we observed the flattening of mucosal folds (Figure 2) which suggests that antiulcerogenic property of AM might be due to a reduction in gastric motility. 

Microscopic evaluation revealed protection of gastric mucosa and decrease in leucocytes infiltration of gastric tissues from AM-pretreated animals as shown in Figure 3. Reduction of neutrophil infiltration into ulcerated gastric endothelia is known to encourage the healing of gastric ulcers in rats. Infiltrated neutrophils produce oxygen free radicals which have inhibitory effect on gastric ulcers healing in rats. These reactive oxygen species are also highly cytotoxic and can provoke tissue damage. Furthermore, neutrophil accumulation in gastric mucosa has been shown to induce microcirculatory abnormalities. Cheng and Koo [25] reported that plant extracts were able to protect gastric mucosa against ethanol induced ulceration through the decrease in neutrophil infiltration. These morphological results were in line with biochemical indicators which revealed that AM has an anti-inflammatory activities. To further confirm this cytoprotective effect, we have evaluated AM against the activity of gastric NO and *in vitro* COX-2. This natural compound was observed to affect the activities of these cellular mediators. NO was reported to inhibit neutrophil infiltration. The role of NO in the protective effect of phytochemicals in ethanol-induced gastric ulcer mice was reported earlier [26]. *α*-Mangostin (AM), was reported to have anti-COX-2 and anti-NO activities [18].

The gastric tissues were also examined for immune-localization of Hsp70 protein to understand the mechanism of AM as an antiulcerogenic agent (Figure 5). Histological examination reveals high numbers of proliferated cell as well as modulation of Hsp70 expression. These results indicate an effective role of this protein in gastroprotection of AM. Hsp70 is the most preserved and richly produced protein in reaction to harmful effects and oxidative stress. Heat shock proteins protect cellular homeostasis from injuries by protecting the structure of normal proteins and repairing or removing damaged proteins. Hsp70 proteins defend cells from oxidative stress or heat shock. Ethanol-generated ROS normally acts to inhibit the expression of Hsp70 [27, 28]. 

Gastric ulcer disease is the most common ulcer of an area of the stomach, and 80% of such ulcers are linked with *Helicobacter pylori*, a spiral-shaped bacterium that lives in the acidic environment of the stomach. *H. pylori *colonizes the stomach and induces long-lasting inflammation (chronic gastritis). The bacterium persists for decades in the stomach. About 10–20% of those colonized by *H. pylori* will eventually experience gastric and duodenal ulcers. *H. pylori* infection is also associated with a 1-2% lifetime risk of stomach cancer and a less than 1% risk of gastric lymphoma. *α*-Mangostin represents minimum inhibitory concentration (MIC) of 25 *μ*g/mL and minimum bactericidal concentration (MBC) of 250 *μ*g/mL against *H. pylori* (Strain no. NCTC11637) and MIC 19 *μ*g/mL and MBC of 125 *μ*g/mL against *H. pylori *(Strain no. J99). *α*-Mangostin was reported before to show an antibacterial activity [29]. 

In conclusion, AM could significantly and dose-dependently protect the gastric mucosa against ethanol-induced injury. The antioxidant activity of this natural compound, through the induction of cellular antioxidant protection, is a pointer for scavenging the free radicals produced by ethanol regardless the weak *in vitro* antioxidant results obtained in this study. The compound also interferes with the natural release of NO and inhibits COXs. The current findings warrant further research for the introduction of AM as a possible defensive and remedial agent for gastric ulcer that is caused by different aetiologies. 

## Figures and Tables

**Table 1:** Observed ulcer area, inhibition percentage pH and mucus content stomach.

Animal group	Pre-treatment (5 mL/kg dose)	pH of gastric content	Mucus content	Ulcer index (mm)^2^ (Mean ± S.E.M)	Inhibition (%)
1	Tween80 (normal control)	3.06^a^ ± 0.09	1.95^a^ ± 0.06		
2	Ethanol 95% (ulcer control)	1.67^b^ ± 0.15	0.99^b^ ± 0.04	482.4^a^ ± 30.89	
3	Omeprazole (20 mg/kg)	3.7^a^ ± 1.14	4.5^c^ ± 0.07	108^b^ ± 9.60	78.42
4	*α*-mangostin (10 g/kg)	4.64^d^ ± 0.1	1.875^d^ ± 0.04	169.92^c^ ± 75.05	66.04
5	*α*-mangostin (30 mg/kg)	3.20^e^ ± 0.05	3.1^e^ ± 0.01	128.16^d^ ± 84.75	74.39

All values are expressed as mean ± standard error of the mean. Means with different superscripts are significantly different. The mean difference is significant at the 0.05 level. Groups with different superscript alphabets are statistically significant.

**Table 2:** Effects of *α*-mangostin (AM) of liver function tests (ALT and AST), gastric MDA, GSH, NP-SH and nitric oxide.

Animal groups	Pre-treatment (5 mL/kg dose)	AST	ALT	GSH	MDA (μmol/g tissue)	NP-SH (μmol/g tissue)	Nitric oxide [NO_2_^−^] μM
1	Tween80 (normal control)	61.06^a^ ± 1.1	145.06^a^ ± 1.8	1.6^a^ ± 0.03	14^b^ ± 0.31	0.6^a^ ± 0.09	9.2^a^ ± 0.19
2	Ethanol 95% (ulcer control)	97^b^ ± 0.15	292.2^b^ ± 19.2	0.7^b^ ± 0.10	28^a^ ± 0.14	7.2^b^ ± 0.5	3.32^b^ ± 0.21
3	Omeprazole (20 mg/kg)	63^a^ ± 0.76	176^a^ ± 4.1	1.7^a^ ± 0.03	15^a^ ± 0.21	1.71^a^ ± 1.4	7.7^a^ ± 1.12
4	*α*-mangostin (10 g/kg)	64^d^ ± 1.5	173^d^ ± 3.6	1.41^d^ ± 0.3	14^d^ ± 0.12	2.6^d^ ± 0.19	6.3^d^ ± 0.14
5	*α*-mangostin (30 mg/kg)	59^e^ ± 6.2	164^e^ ± 5.0	1.7^e^ ± 0.02	14^e^ ± 0.11	1.40^e^ ± 0.93	8.1^e^ ± 0.53

Values are presented as mean ± SEM of five rats in each group. Glutathione (GSH) levels, malondialdehyde level (MDA), nitric oxide (NO), alanine transaminase (ALT), aspartame transaminase (AST) and non-protein sulfhydryl groups (NP-SH) were measured in all groups. Groups with different alphabets are statistically different.
